# MicroRNA-219 Inhibits Proliferation and Induces Differentiation of Oligodendrocyte Precursor Cells after Contusion Spinal Cord Injury in Rats

**DOI:** 10.1155/2019/9610687

**Published:** 2019-02-18

**Authors:** Fang Li, Mou-Wang Zhou, Nan Liu, Yan-Yan Yang, Hua-Yi Xing, Yao Lu, Xiao-Xie Liu

**Affiliations:** Department of Rehabilitation Medicine, Peking University Third Hospital, 49 North Garden Road, Beijing 100191, China

## Abstract

MicroRNA-219 (miR-219) regulates the proliferation and differentiation of oligodendrocyte precursor cells (OPCs) during central nervous system (CNS) development. OPCs only differentiate into oligodendrocytes (OLs) in the healthy CNS, but can generate astrocytes (As) after injury. We hypothesized that miR-219 may modulate OPC proliferation and differentiation in a cervical C5 contusion spinal cord injury (SCI) model. After injury, we observed a decrease in the miR-219 level and quantity of OLs and an increase in the number of OPCs and As. Silencing of miR-219 by its antagomir *in vivo* produced similar results, but of greater magnitude. Overexpression of miR-219 by its agomir *in vivo* increased the number of OLs and suppressed generation of OPCs and As. Luxol fast blue staining confirmed that SCI caused demyelination and that the extent of demyelination was attenuated by miR-219 overexpression, but aggravated by miR-219 reduction. Monocarboxylate transporter 1 (MCT-1) may be implicated in the regulation of OPC proliferation and differentiation mediated by miR-219 following contusion SCI. Collectively, our data suggest that miR-219 may mediate SCI-induced OPC proliferation and differentiation, and MCT-1 may participate in this process as a target of miR-219.

## 1. Introduction

Spinal cord injury (SCI) is a common and serious injury of the central nervous system (CNS) typically resulting in sustained sensorimotor dysfunction and can severely affect patients' quality of life [1]. Pathophysiologically, SCI involves both primary neural injury and secondary tissue damage. Primary injury is caused by initial mechanical change. Secondary damage is induced by vascular and biochemical changes and leads to oligodendrocyte death and axon demyelination, which may leave axons vulnerable to degeneration. Targeting remyelination of axons therapeutically to promote functional benefits is considered a potential treatment strategy after SCI [1–3].

Mature oligodendrocytes (OLs) are the sole myelinating cells of the CNS. OLs support axons and maintain neurological function. The death of OLs after SCI leads to demyelination and thereby exacerbates neurological deficits. Surviving OLs after injury are postmitotic and unable to contribute to cell renewal for generating more myelin. New myelinating OLs are solely derived from oligodendrocyte precursor cells (OPCs), which are abundantly expressed throughout the life span throughout the entire CNS [4–6]. OPCs, also known as NG2 glia or vascular pericytes, are considered the fourth glial population in addition to astrocytes (As), OLs, and microglia, since a significant portion of them persists in the adult CNS [7, 8]. OPCs of the healthy spinal cord exist in a low proliferation state and only differentiate into OLs. They do not generate As, but in the injured spinal cord, OPCs extensively proliferate, gain a more plastic fate, and generate As [7, 9–11].

MicroRNAs (miRNAs) are a class of small (19-24 nucleotides) noncoding RNAs that mediate posttranscriptional regulation of target genes by translational repression or promoting RNA degradation and act as important regulators during disease progression and recovery [12]. Several reports indicate that hosts of miRNAs such as miR-219, miR-338, and miR-138 are critical for CNS development and physiology, with roles in OPC proliferation and differentiation [13–15]. Among these miRNAs, miR-219 is necessary and sufficient to modulate OPC proliferation and differentiation [16, 17]. However, whether miR-219 regulates SCI-induced OPC proliferation and differentiation has not been reported.

Monocarboxylate transporter 1 (MCT-1) is predominantly expressed by OLs in the CNS [18]. It is an important protein that transfers lactate from OLs to axons, which ensures that the CNS can effectively use lactate to obtain enough energy when glucose is insufficient *in vivo* [19]. It is of great significance to the energy metabolism of the CNS. Liu et al. revealed that MCT-1 is involved in the differentiation of OPCs induced by miR-219 [20]. Thus, we hope to further explore whether MCT-1 is related to the role of miR-219 in regulation OPC proliferation and differentiation after SCI.

The specific animal model employed is crucial for our study. Given that 51% of SCI patients sustain injuries to the cervical spine, with the most common neurological level being C5 followed by C4 and C6 [21]. Indeed, contusion injury is the most clinically relevant type of SCI [22, 23]. Moreover, the degree of demyelination that occurs in contusion injury is the most severe [24, 25]. Therefore, in the present study, we used a cervical C5 unilateral contusion model to investigate the effects of miR-219 on OPC proliferation and differentiation. Our data show for the first time that miR-219 inhibits proliferation and promotes differentiation of OPCs, partially improves forelimb function, and enhances myelin repair in a contusion SCI model.

## 2. Materials and Methods

### 2.1. Animals and Grouping

All animal experiments were performed in accordance with the recommendations of the Chinese Laboratory Animal Requirements of Environment and Housing Facilities. The procedures were approved by the Committee on the Ethics of Animal Experiments of Peking University. A total of 160 male Sprague-Dawley rats (6-8 weeks of age, 180-220 g) were purchased from the Experimental Animal Center of Peking University Health Science Center. The rats were housed under controlled environmental conditions (22°C with alternating 12 h light and dark cycles) and received standard rat chow and water *ad libitum*. The animals were randomly divided into six groups: sham (*n* = 10), SCI (*n* = 30), SCI + agomir-219 (*n* = 30), SCI + agomir-negative control (NC) (*n* = 30), SCI + antagomir-219 (*n* = 30), and SCI + antagomir-NC (*n* = 30). All groups, with the exception of the sham group, were subdivided into five time points: 1-, 3-, 7-, 10-, and 14-day post-SCI (*n* = 6 per time point). Several cohorts were studied due to the size of the experiment, but care was taken to ensure that equal numbers of animals from each group were studied in each cohort. All animals survived the experimental period without adverse effects and were included in the data analysis.

### 2.2. Cervical SCI Model

Surgical procedures were performed as described previously [22]. In brief, the rats were anesthetized by intraperitoneal injection with 2% sodium pentobarbital (0.3 mL per 100 g body weight). The neck region was shaved and aseptically prepared for surgery. A dorsal midline incision was made to expose the C4-C6 vertebrae. A C5 unilateral laminectomy was performed on the dominant side, to allow the passage of an impactor tip of 1.5 mm in diameter. The animal was mounted within a clamping system that was developed to firmly grasp and stabilize the spinal column from C4 to C6. A spinal cord contusion injury was created using an Infinite Horizon spinal cord impactor device (IH impactor; Precision Systems and Instrumentation, Lexington, KY, USA) with a preset force of 150 kdynes. The sham group only underwent laminectomy. Subsequently, the vertebral clamps were removed. The skin and muscle were sutured to promote healing and avoid infection. Immediately after surgery and twice daily (a.m. and p.m.) for a 3-day post-SCI, the rats received subcutaneous injections of Ringer's sodium lactate solution (5 mL) and sodium ampicillin (80 mg/kg; Harbin Pharmaceutical Group Co. Ltd., China).

### 2.3. Injection of miR-219

The miR-219-related products were injected in the following four groups: the SCI + agomir-219 (agomir-219) group (RiboBio, Guangzhou, China), the SCI + agomir-NC (agomir-NC) group (RiboBio), the SCI + antagomir-219 (antagomir-219) group (RiboBio), and the SCI + antagomir-NC (antagomir-NC) group (RiboBio). The rats were subjected to SCI and then received injections of agomir-219 (1 nmol/100 g body weight, 0.5 nmol/*μ*L), agomir-NC (1 nmol/100 g body weight, 0.5 nmol/*μ*L), antagomir-219 (2 nmol/100 g body weight, 1 nmol/*μ*L), or antagomir-NC (2 nmol/100 g body weight, 1 nmol/*μ*L) in the lesion epicenter using a microinjection pump (Beijing Zhongshang Boao Biotechnology Co. Ltd.) and Neuros syringes (65460-05, Hamilton Company, Switzerland) starting 15 min after contusion SCI. The injection rate was 0.2 *μ*L/min, and the needle was left in place for an additional 5 min before being slowly withdrawn. The volumes and concentrations of miR-219 were determined based on a preexperimental result and the manufacturer's protocol.

### 2.4. Bromodeoxyuridine (BrdU) Administration

To label proliferating cells, the thymidine analog, bromodeoxyuridine (BrdU; 10 mg/mL in sterile saline; Sigma-Aldrich, St. Louis, MO, USA) was injected intraperitoneally (100 mg/kg) once daily. Rats in each group were given a specific BrdU pulsing regimen as outlined in Table 1 [26].

### 2.5. Behavioral Assessments

All behavioral assessments were conducted and quantified by researchers blind to the experimental treatments.

#### 2.5.1. Cylinder Rearing Test

Animals were tested before injury and at 1, 3, 7, 10, and 14 days postoperatively. The purpose of preinjury assessment was to determine the dominant sides of the rats and to damage the superior sides of the spinal cords. Details on the cylinder rearing test have been described previously [27–29]. Briefly, the rats were placed in a topless clear plexiglass cylinder for 15 min. Two mirrors were placed at 90° angles behind the cylinder such that the forelimbs could be viewed at all times. The animals were videotaped during spontaneous vertical exploration, and a frame-by-frame analysis of the forelimb usage in 20 wall-touching rears was scored by coauthors who were blind to the grouping. During a rearing motion, paw usage of the affected forelimb was scored as “ipsilateral only” or “both (ipsi + contra)” usage.

#### 2.5.2. Grooming Test

The grooming test was performed preoperatively to obtain baseline scores and at 1, 3, 7, 10, and 14 days postsurgery. The animals were placed in a clear cylinder with mirrors at angles. A few drops of cool saline were applied to the rats' head and back with soft gauze. The testing session was videotaped for 15 min. Frame-by-frame video playback was used to score each forelimb independently by the maximal range of motion during grooming. The detailed scoring system was as follows [30]: 0, the animal was unable to contact any part of the face or head; 1, the animal's forepaw touched the underside of the chin and/or the mouth area; 2, the animal's forepaw contacted the area between the nose and the eyes, but not the eyes; 3, the animal's forepaw contacted the eyes and the area up to, but not including, the front of the ears; 4, the animal's forepaw contacted the front but not the back of the ears; 5, the animal's forepaw contacted the area of the head behind the ears.

### 2.6. Tissue Processing

According to the scheduled time point, animals were euthanized with an overdose of sodium pentobarbital. Some rats were transcardially perfused with 150 mL of sterile saline followed by 300 mL of 4% paraformaldehyde (Coolaber, Beijing, China). A length of spinal cord between C2 and T2 was removed, postfixed in 4% paraformaldehyde at 4°C for 6 h, and then cryoprotected in 10%, 20%, and 30% sucrose (in phosphate-buffered saline (PBS)) overnight, respectively. Spinal cord sections of 1 cm in length containing the injury site were quickly embedded in optimal cutting temperature compound (Sakura, USA) and fast frozen in liquid nitrogen. The spinal cords were cross sectioned at 14 *μ*m thickness and stored at −80°C (note: it was difficult to successfully remove a complete frozen spinal cord section containing the lesion site due to the apparent congestion of the injury region at the time point of a 1-day postsurgery. Therefore, pathological examination was not performed at a 1-day postsurgery). Other samples measuring 8 mm in length were directly removed from fresh spinal cords containing the lesion epicenter and were immediately stored in liquid nitrogen for subsequent molecular biology experiments.

### 2.7. Quantitative Real-Time PCR (qRT-PCR)

Total RNA was extracted from spinal cord tissues in TRIzol (Invitrogen, Thermo Fisher Scientific Inc., USA) following the manufacturer's protocol. To generate cDNA for the measurement of miR-219 levels, total RNA (2 *μ*g/sample) was reacted with a miRcute-enhanced miRNA cDNA first-strand synthesis kit. Subsequently, the expression levels of miR-219 were analyzed using a miRcute-enhanced miRNA fluorescence detection kit. U6 was used as an internal control. Alternatively, to produce cDNA for the analysis of the expression of other genes, total RNA (2 *μ*g/sample) was reacted with a fastking cDNA first-strand synthesis kit followed by incubation with a talent qPCR premix fluorescence detection kit. The expression level of glyceraldehyde 3-phosphate dehydrogenase (GAPDH) was used as the internal control. All of the above kits were purchased from Tiangen Biochemical Technology (Beijing) Co. Ltd. All primers used in this experiment were provided by Sangon Biotech (Shanghai) Co. Ltd. The QuantStudio Design and Analysis software (Applied Biosystems) was used to determine the cycle threshold (CT) fluorescence values. The data were analyzed using the 2^-ΔΔCT^ method by a person who was blind to the treatment groups of each animal (Table 2 lists the primer sequences).

### 2.8. Western Blotting

Total protein was extracted using the radio immunoprecipitation assay (RIPA) lysis buffer (including a protease inhibitor cocktail) [1]. After the concentration of proteins was measured using a BCA assay kit (Thermo Scientific, MA, USA), they were subjected to western blotting. Equal amounts of proteins were separated using 10% SDS-PAGE and transferred to a nitrocellulose membrane (Applygen Technologies, Beijing, China). The membrane was blocked with 5% nonfat milk for 1 h at room temperature (RT, 22-25°C) and then incubated with primary antibodies including rabbit anti-MCT-1 antibody (1 : 500; Millipore, CA, USA) and mouse anti-GAPDH antibody (1 : 10000; Abcam, Cambridge, UK) at 4°C overnight. The membrane was incubated with IRDye 800CW-conjugated antibodies for 1 h, and the target proteins were scanned on a LI-COR Odyssey® Imaging System (Lincoln, NE, USA). The density of the products was quantified using the ImageJ software (NIH, Bethesda, MD, USA) by two examiners who were blind to the identity of the samples being studied.

### 2.9. Immunofluorescence

The sections were thawed at RT for 30 min, washed three times (5 min each) in 1 × PBS with 0.1% Triton X-100 (Beyotime Biotechnology, Shanghai, China), and placed in a block solution consisting of 10% normal goat serum (Zhongshan Golden Bridge, Beijing, China)/0.1% Triton X-100/1 × PBS for 1 h. The sections were then incubated with rabbit antimyelin basic protein (MBP) to identify mature OLs (1 : 200; Cat no. ab40390; Abcam, Cambridge, UK) or mouse antiglial fibrillary acidic protein (GFAP) to identify As (1 : 200; Cat no. ab10062; Abcam, Cambridge, UK) overnight at 4°C. Following incubation in the primary antibodies, the sections were washed again with 1 × PBS/0.1% Triton X-100 and incubated for 1 h at RT with the respective fluorescent secondary antibodies: Alexa Fluor 594-labeled anti-rabbit IgG (1 : 100; Cat no. ZF0516; Zhongshan Golden Bridge, Beijing, China) or Alexa Fluor 488-labeled anti-mouse IgG (1 : 100; Cat no. ZF0512; Zhongshan Golden Bridge, Beijing, China). Subsequently, the nuclei were stained with 4′,6-diamidino-2-phenylindole (DAPI) (Beyotime Biotechnology, Shanghai, China) and then mounted. Images were captured using a confocal microscope (Leica, SP8, Germany).

For double-immunofluorescent labeling with NG2 and BrdU, sections were thawed and rinsed as above. The sections were then suspended in HCl (1 N) for 10 min on ice to lyse the DNA structure of the labeled cells. This was followed by suspension in HCl (2 N) for 10 min at RT before incubation for 20 min at 37°C. Immediately after incubation in the acid, borate buffer (0.1 M) was used to buffer the sections for 12 min at RT, followed by washing and blocking for 60 min with 5% normal goat serum/1 M glycine (AMRESCO, USA)/0.1% Triton X-100/1 × PBS. The sections were incubated overnight at 4°C with a combination of mouse anti-BrdU (1 : 500; Cat no. 11202693001; Roche, USA) and rabbit anti-NG2 (1 : 200; Cat no. AB5320; Millipore, CA, USA). The remaining stain was conducted as per the immunostaining protocol described above.

For quantification, three animals per group were examined at each time point. The number of BrdU+ with NG2+ and GFAP+ cells in the lesion regions of the spinal cord was measured in three sections per animal from the same levels. Four fields of the view per section were randomly imaged under a 20x objective magnification. The numbers of immunoreactive cells were presented as numbers per mm^2^. For quantitative analysis of OLs, we calculated average optical density of the MBP immunofluorescence staining per field. The reason is that the level of MBP expression reflects the number of myelinating OLs [31] (average optical density = integral optical density (IOD)/area). Experimenters performing the analysis were blinded to the group identities of the rats.

### 2.10. Luxol Fast Blue (LFB) Staining

Luxol fast blue (LFB) staining for myelin was used in 14 *μ*m thick sections. The cryostat sections were processed according to the cryo-nerve myelin fast blue staining kit protocol (Genmed Scientifics Inc., USA). We adopted the experimental protocol as reported previously [32]. Images were captured under a NanoZoomer Digital Pathology (Hamamatsu). Three blinded experimenters calculated the staining density to quantify the myelin using the Image J [33]. Three animals per group were examined at each time point. Three spinal cord cross sections per animal from the same levels in the injured regions were analyzed.

### 2.11. Statistical Analysis

Statistical analyses were conducted using the SPSS 20.0 software (Illinois, USA). All data are expressed as mean ± standard error of the mean (SEM). The assumption of normality and homogeneous variance were verified for continuous variables before conducting any group comparisons. The comparison among groups was performed using one-way analysis of variance (ANOVA). A *p* value of <0.05 was considered statistically significant.

## 3. Results

The animals underwent cervical C5 unilateral contusion injuries with an average contusive compressive force of 152.848 ± 0.483 kdynes, which was close to the preset contusive peak force of 150 kdynes. This slight difference was attributable to the inertial compensation with a blank hit. In addition, there were no deaths during or after any of the surgical operations, and no animals exhibited signs of blood in their urine or stool.

### 3.1. SCI Downregulates miR-219 Expression

**Figure 1:**
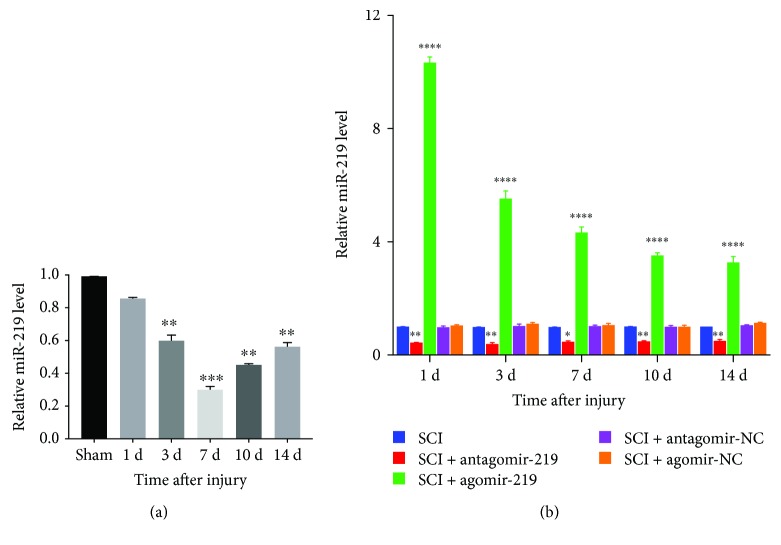
Expression of miR-219 in different groups. (a, b) Relative expression level of miR-219 detected by qRT-PCR over the time course in all groups. Each bar represent the means ± SEM for triplicate experiments. ^∗^*p* < 0.05, ^∗∗^*p* < 0.01, ^∗∗∗^*p* < 0.001, and ^∗∗∗∗^*p* < 0.0001. NC: negative control.

The expression of miR-219 at different time points in all groups was quantified using qRT-PCR. The expression level of miR-219 on days 3, 7, 10, and 14 of postsurgery in the SCI group was significantly lower than that in the sham group and reached the lowest level on day 7 after SCI. The miR-219 expression levels on day 1 did not significantly differ (Figure 1(a)). We overexpressed and inhibited miR-219 function in SCI rats. The qRT-PCR results demonstrated that miR-219 expression was increased over the time course in the agomir-219 group and was downregulated in the antagomir-219 group. There was no difference in the miR-219 expression levels between the agomir-NC and SCI groups. Similarly, the miR-219 expression levels in the antagomir-NC and SCI groups were not significantly different (Figure 1(b)).

### 3.2. MiR-219 Regulates OPC Proliferation and Differentiation following Contusion SCI

OPCs only differentiate into OLs under physiological conditions, but generate As after SCI [7, 11]. MiR-219 promotes cell cycle arrest and differentiation of OPCs during CNS development [17, 34–36]. We hypothesized that miR-219 would inhibit the proliferation of OPCs and promote the differentiation of OPCs into OLs after contusion SCI. We first examined the relationship between miR-219 and OPCs following contusion SCI.

**Figure 2:**
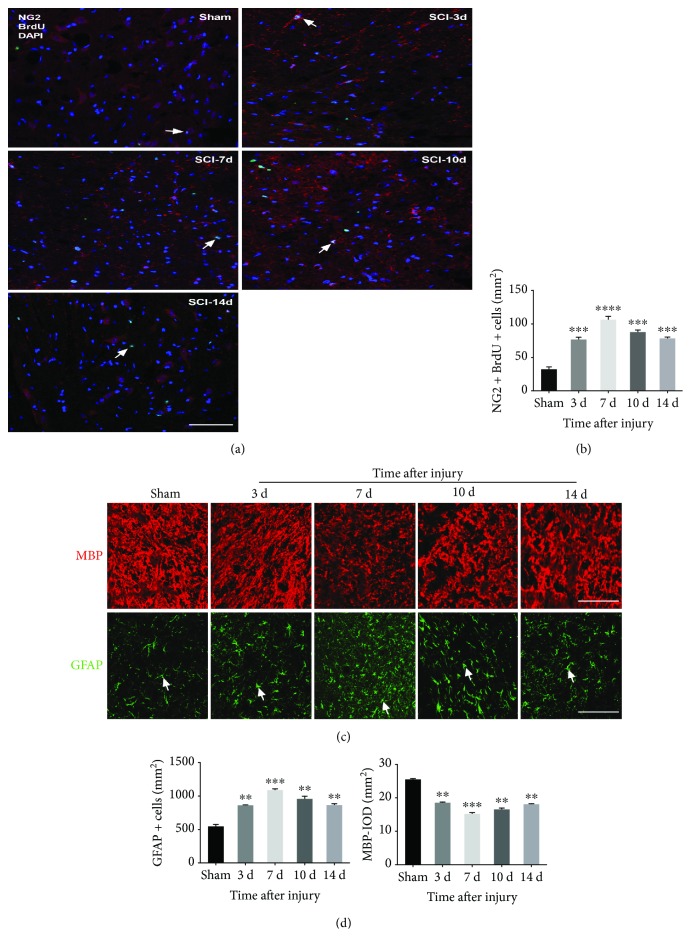
Dynamic changes in NG2+ with BrdU+ OPCs, MBP+ oligodendrocytes, and GFAP+ astrocytes after contusion SCI. (a, c) Representative immunostained images of NG2 positive (red) with BrdU positive (green) cells, MBP positive (red) cells, and GFAP positive (green) cells, respectively, between the sham and SCI group. (b, d) Quantitative data of NG2+ with BrdU+ OPCs, MBP+ oligodendrocytes, and GFAP+ astrocytes, respectively, between the sham and SCI group. ^∗∗^*p* < 0.01, ^∗∗∗^*p* < 0.001, and ^∗∗∗∗^*p* < 0.0001. Scale bar = 50 *μ*m. OPCs: oligodendrocyte precursor cells; MBP: myelin basic protein; GFAP: glial fibrillary acidic protein; BrdU: bromodeoxyuridine.

We used immunofluorescence to detect changes in NG2+/BrdU+ OPCs (proliferating OPCs), MBP+ OLs, and GFAP+ As at 3, 7, 10, and 14 days after contusion SCI. We observed that the number of proliferating OPCs and As at days 3, 7, 10, and 14 was significantly higher in the SCI group than in the sham group, whereas the number of OLs was reduced. At 7 days after SCI, the numbers of proliferating OPCs and As were the highest, and the number of OLs was the lowest (Figure 2).

**Figure 3:**
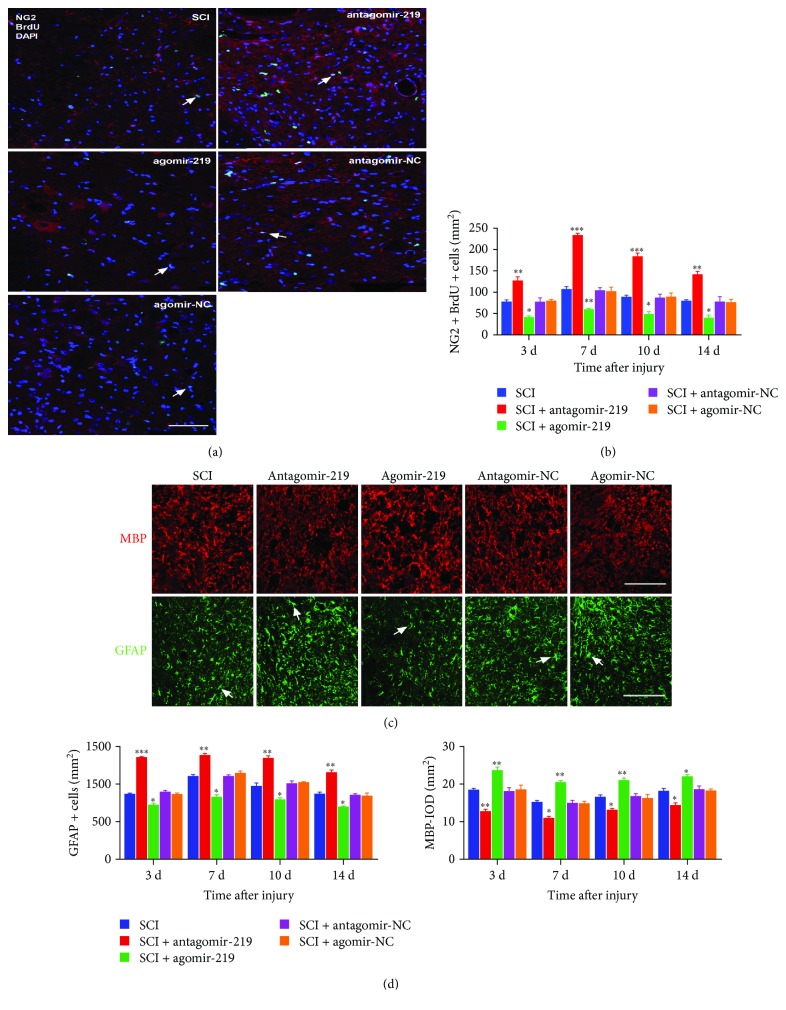
The effects of miR-219 on the proliferation and differentiation of OPCs (typical images at 7 days). (a, c) Typical images of NG2+/BrdU+ OPCs, MBP+ oligodendrocytes, and GFAP+ astrocytes in the SCI, antagomir-219, agomir-219, antagomir-NC, and agomir-NC groups, with NG2 (red), BrdU (green), DAPI (blue), MBP (red), and GFAP (green). (b, d) The quantitative data of NG2+/BrdU+ OPCs, MBP+ oligodendrocytes, and GFAP+ astrocytes, respectively, at days 3, 7, 10, and 14 in the SCI, antagomir-219, agomir-219, antagomir-NC, and agomirNC groups. ^∗^*p* < 0.05, ^∗∗^*p* < 0.01, and ^∗∗∗^*p* < 0.001. Scale bar = 50 *μ*m. OPCs: oligodendrocyte precursor cells; MBP: myelin basic protein; GFAP: glial fibrillary acidic protein; BrdU: bromodeoxyuridine; NC: negative control.

Furthermore, to examine the effect of miR-219 on OPC proliferation and differentiation, we performed overexpression and inhibition of miR-219 in rats with contusion SCI. We detected the dynamic changes in proliferating OPCs, OLs, and As at all time points by immunofluorescence. The results revealed that elevation of miR-219 *in vivo* resulted in a significant increase in the number of OLs and a decrease in the number of proliferating OPCs and As (Figure 3). In contrast, attenuation of endogenous miR-219 expression blocked OPCs from differentiating into mature OLs and promoted differentiation into As and induced OPC proliferation, as evaluated by immunofluorescence analysis (Figure 3). These data suggest that miR-219 may restrain OPC proliferation and play an instructive role in promoting OPC differentiation after contusion SCI.

### 3.3. MiR-219 Promotes Myelin Repair after Contusion SCI

**Figure 4:**
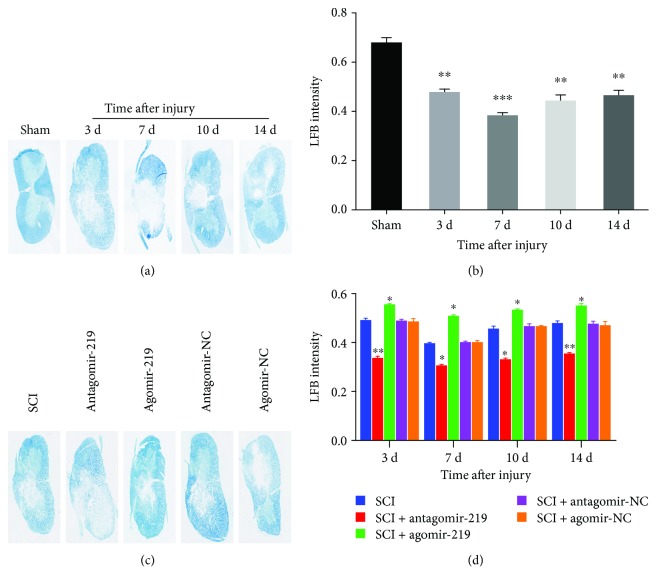
Typical images of Luxol fast blue (LFB) staining for assessment of myelination following contusion SCI. (a) LFB staining for evaluation of myelination indicated demyelination after contusion SCI. (c) The results of LFB staining revealed partial restoration of myelination in animals treated with agomir-219 and aggravated demyelination in the antagomir-219 group (representative images at 3 days after SCI). In addition, no significant group differences were observed between the antagomir-NC or agomir-NC and SCI groups. (b, d) Quantification results of LFB staining. ^∗^*p* < 0.05, ^∗∗^*p* < 0.01, and ^∗∗∗^*p* < 0.001. NC: negative control.

In order to evaluate the effect of miR-219 on myelin repair, we performed specific staining using LFB to determine overall myelination. First, we tested the changes in myelin after contusion SCI. We observed that there was a lower myelination level at all time points in the SCI group compared to the sham group, and myelination reached the lowest level at 7 days (Figures 4(a) and 4(b)). We then evaluated the level of myelination after overexpression and reduction of miR-219 in contusion SCI rats. LFB staining revealed an enhanced myelination level in the agomir-219 group compared to the SCI group. Contrary to the results observed in the agomir-219 group, we observed a significantly lower myelination level in the antagomir-219 group than that in the SCI group. No significant group differences were observed between the antagomir-NC or agomir-NC and SCI groups (Figures 4(c) and 4(d)). These data imply that miR-219 promotes myelin repair after contusion SCI in rats.

### 3.4. MiR-219 Augments the Recovery of Forelimb Motor Function in Contusion SCI Rats

**Figure 5:**
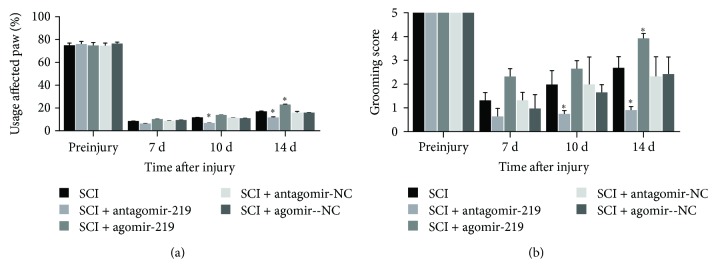
Effect of miR-219 on forelimb movement during behavioral tests after C5 hemicontusion. (a) Compared to preinjury, the rats in the SCI group produced marked deficits in rearing performance on days 7, 10, and 14 after SCI (*p* < 0.001). Rats on days 10 and 14 after SCI in the antagomir-219 group exhibited a more profound decline in usage of the affected paw, whereas a significant increase on day 14 after SCI was observed in the agomir-219 group. (b) A more severe grooming deficit of the ipsilateral paw was observed after contusion injury (*p* < 0.001). At days 10 and 14 postinjury, animals in the antagomir-219 group achieved lower scores than did those in the SCI group, whereas only on day 14 postinjury the rats in the agomir-219 group achieved higher scores than did the SCI group. Error bars indicate SEM. ^∗^*p* < 0.05. NC: negative control.

Of note, at 1 and 3 days after contusion SCI, the rats in all groups aside from the sham group could not complete the cylinder rearing and grooming tests due to surgical injury. The cylinder rearing task is effective at examining a rodent's spontaneous forelimb use for postural support and vertical exploration [37]. Before injury, all rats in all groups used the preferred (ipsilateral) forelimb in 76% of weight support wall-touching in the cylinder rearing test. Compared to preinjury levels, the SCI group displayed a profound reduction in ipsilateral forelimb usage during rearing events (Figure 5(a)). Animals in the antagomir-219 group demonstrated worse rearing performance than did the SCI group; at 10 and 14 days, the differences were significant (Figure 5(a)). An improvement was noted in the usage of the ipsilateral forelimb in the agomir-219 group compared to the SCI group, and at day 14, there was a significant difference (Figure 5(a)). However, neither the antagomir-NC group nor the agomir-NC group exhibited a change in usage of the impaired forelimb compared to the SCI group (Figure 5(a)).

The grooming test was used to evaluate forelimb grooming function based on the ability of the animals to contact the forepaw with any part of the face or head after water was applied to their head and back [38]. In the grooming test, rats in the SCI group achieved lower grooming scores after surgery than before surgery (Figure 5(b)). In the antagomir-219 group, the grooming deficits were more pronounced than those in the SCI group, and there were significant differences at 10 and 14 days after SCI (Figure 5(b)). For the agomir-219 group, although the rats achieved higher grooming scores than did the SCI group, there was a significant difference only at 14 days after SCI (Figure 5(b)). Similar to the results of the cylinder rearing test, the grooming scores were not significantly different between the antagomir-NC or agomir-NC and the SCI groups (Figure 5(b)).

In summary, the results of rearing and grooming tests showed that miR-219 may increase ipsilateral forelimb usage and forepaw range of motion in contusion SCI rats. In other words, miR-219 may promote the recovery of forelimb motor function in rats with contusion SCI.

### 3.5. MCT-1 May Mediate the Effects of miR-219 on OPC Differentiation

**Figure 6:**
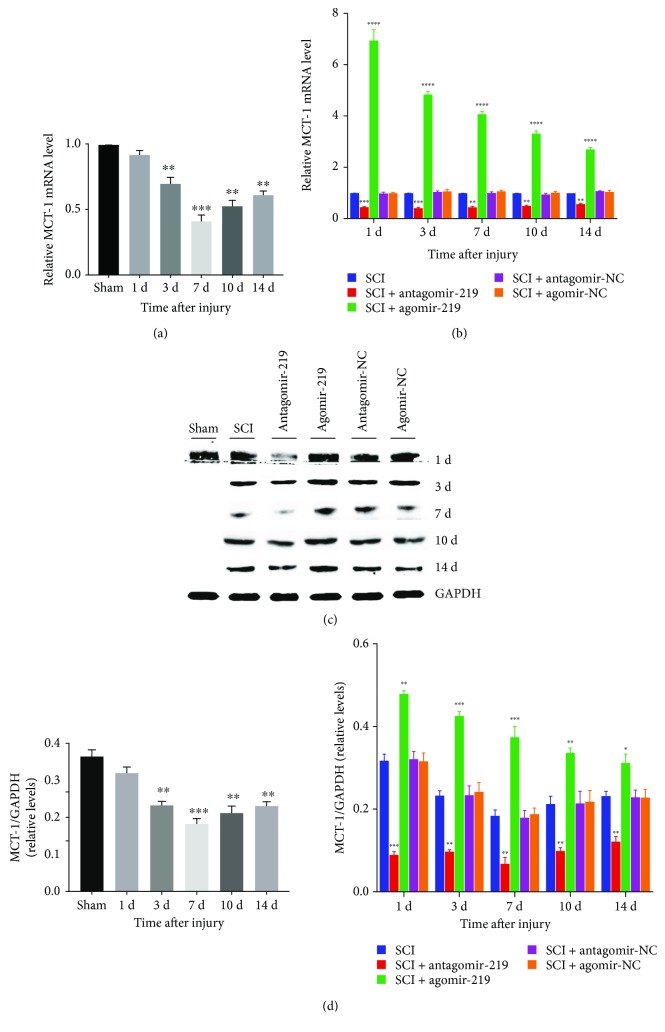
Expression of MCT-1 in different groups. (a, b) Relative expression level of MCT-1 mRNA detected by qRT-PCR over the time course in all groups. (c, d) MCT-1 protein level of different groups at all time points was detected by western blotting. Each bar represent the means ± SEM for triplicate experiments. ^∗^*p* < 0.05, ^∗∗^*p* < 0.01, ^∗∗∗^*p* < 0.001, and ^∗∗∗∗^*p* < 0.0001. MCT-1: monocarboxylate transporter 1; NC: negative control.

A previous study indicated that MCT-1 was crucial for OL maturation and myelin synthesis [39]. Another study suggested that MCT-1 may be associated with the effects of miR-219 on OPC differentiation in cuprizone-induced demyelinated mice [20]. We hypothesized that MCT-1 would be similarly involved after SCI. First, we evaluated the expression of *MCT-1* gene by qRT-PCR. The results revealed that it was regulated in a similar manner to miR-219 (Figures 6(a) and 6(b)). Furthermore, the protein expression of MCT-1 was examined in different groups by western blotting. We observed that alteration of the MCT-1 protein was also in parallel with that of miR-219 (Figures 6(c) and 6(d)).

The above data demonstrate that the expression levels of *MCT-1* mRNA and MCT-1 protein on days 3, 7, 10, and 14 after SCI were all significantly lower in the SCI group than in the sham group, whereby they were lowest on day 7 after SCI. There was no significant difference in the *MCT-1* mRNA and MCT-1 protein expression levels on day 1 after SCI. Agomir-219 reversed the decrease in MCT-1 induced by SCI, and antagomir-219 exacerbated the reduction of MCT-1 caused by SCI. However, neither the agomir-NC nor the antagomir-NC affected the change in MCT-1 induced by SCI. This finding suggested that miR-219 enhanced MCT-1 expression, and MCT-1 may be an indirect target of miR-219 after SCI. However, more detailed and comprehensive experiments are required to validate these preliminary findings.

## 4. Discussion

Several studies have reported that expression of miR-219 is decreased after CNS injury, but how the expression level of miR-219 is affected by SCI, in particular at the acute and subacute SCI phases, remains unclear [40–42]. Here, we present evidence that the level of miR-219 was reduced at the lesion center of the injured spinal cord tissues in the acute and subacute SCI phases, with the lowest level of miR-219 expression occurring on day 7 after injury. Furthermore, we microinjected agomir-219 and antagomir-219 at the injury site to overexpress and reduce the expression level of miR-219, respectively, *in vivo*. Detection by qRT-PCR indicated that miR-219 overexpression and reduction at all time points were successful. However, the current mainstream method of overexpression and silencing *in vivo* and *in vitro* is viral transfection, which does not cause secondary damage, but is often accompanied by adverse reactions and low transfection efficiency. Although the method of microinjection at the injury site is less widely used, it achieves a higher drug concentration at the site of injury and thus exerts a longer effective action time [43].

Previous studies have reported that OPCs are restricted to become OLs during spinal cord development, but give rise to As and OLs in the spinal cord after SCI [7, 44]. Several other studies have confirmed that miR-219 plays an important role in OPC proliferation and differentiation and promotes myelination in the normal CNS and a multiple sclerosis animal model [17, 20, 36]. Although previous reports have indicated that miR-219 is implicated in the positive control of OPC differentiation, we demonstrated for the first time that miR-219 inhibited proliferation and promoted differentiation of OPCs following SCI, which in this study was a model of cervical C5 hemicontusion. Through a series of immunostainings for related cell markers (NG2, MBP, and GFAP) and the proliferation marker, BrdU, we observed an increase in the number of proliferating OPCs and As, but a decrease in the number of OLs after injury. This may be related to the decreased expression level of miR-219 after injury. Moreover, we found that miR-219 upregulation could promote the generation of OLs and decrease the number of proliferating OPCs and As. Attenuation of miR-219 expression elicited the opposite effect to miR-219 upregulation. Additionally, the presence of demyelination after injury, remyelination induced by miR-219 overexpression, and the degree of demyelination aggravated by miR-219 downregulation were confirmed by LFB staining [33, 45]. Collectively, our findings support previous results indicating that OPCs not only generate OLs but also give rise to As after SCI. We also demonstrated that miR-219 inhibited the proliferation of OPCs and induced beneficial differentiation of OPCs into OLs after injury. In addition, we demonstrated that miR-219 promoted myelin repair following contusion SCI. These results provide insight into the potential roles of miR-219 in the regulation of OPC proliferation and differentiation, as well as remyelination after contusion SCI.

However, although our study and multiple previous publications pointed out OPCs could differentiate into OLs and As after injury, recent evidence suggested that OPCs also generated Schwann cells, as well as both OLs and Schwann cells contributed to the myelination of axons after SCI [3, 46]. In this setting, Schwann cells also produce MBP which is a major constituent of the myelin sheath [47]. The expression level of MBP can reflect the number of myelinating OLs in the normal CNS, but may indicate the total number of OLs and Schwann cells after CNS damage [31]. Considering Schwann cells might mediate remyelination of injured spinal axons, our analytical approach that average optical density of the MBP stained area represented the number of OLs after SCI showed certain limitation. For this reason, in our further research, we would explore the detailed and comprehensive differentiation of OPCs after cervical C5 unilateral contusion in rats where we would employ a true mature OL marker like CC1 or GSTpi to look at OLs [48, 49].

Additionally, LFB staining has certain specificity and is a good staining method for identifying myelin, but it cannot distinguish old myelin from new myelin post-SCI. Therefore, the underlying mechanism of the effect that miR-219 can promote myelin repair observed by LFB staining after SCI is enhancing remyelination or protecting myelin/OLs from degeneration. To better explain the effect of miR-219 on myelin following SCI, we would analyze myelin by LFB staining and electron microscopy in later work.

We used the cylinder rearing task and grooming test to assess forelimb function and observed that most tasks for the ipsilateral forelimb were abolished after injury [27, 38]. There was a significant reduction in the range of motion using the ipsilateral forepaw and less ipsilateral paw usage in the grooming and rearing tests after injury. The present study also observed stable forelimb deficit throughout the entire experiment in the SCI group, but the deficit was less severe than the behavioral impairments reported in previous studies using the same injury model [22, 28, 45]. A possible explanation is that the rats used in our study were younger, which may result in more rapid recovery. We observed that agomir-219 enhanced the usage of the ipsilateral forelimb and increased the range of motion in the ipsilateral forepaw from 14 days after SCI. However, antagomir-219 worsened this performance from 10 days following SCI. A possible explanation is that agomir-219 and antagomir-219 may function differently *in vivo*. These data suggest that miR-219 may be critical for recovery of behavioral function in SCI rats.

MCT-1, also referred to as SLC16A1, MCT-2, and MCT-4 are extracellular membrane proteins that facilitate the transport of monocarboxylic acids such as lactate, pyruvate, and ketone bodies across biological membranes to produce ATP to match particular metabolic needs in the CNS [20, 28, 50]. MCT-1 is predominantly expressed in oligodendroglia [18]. One study proposed that miR-219 attenuated demyelination in cuprizone-induced demyelinated mice by regulating MCT-1 [20]. Therefore, we hypothesized that miR-219 may regulate the proliferation and differentiation of OPCs by targeting MCT-1, thereby promoting remyelination after contusion SCI. Thus, we tested the changes in MCT-1 expression in contusion SCI rats. The results indicated that the expression of MCT-1 was downregulated in the injured spinal cord, and that miR-219 upregulation reversed the decrease in MCT-1 induced by contusion SCI. Conversely, silencing miR-219 exacerbated the reduction of MCT-1 after contusion SCI. Based on the immunofluorescence results, we propose that miR-219 regulates OPC proliferation and differentiation by indirectly targeting MCT-1 in a rat contusion SCI model. However, it remains unclear whether the miR-219-associated increase in MCT-1 is related to its role in OPC proliferation and differentiation. Further research is necessary to clarify the precise regulatory roles of miR-219 and MCT-1 in OPC proliferation and differentiation following SCI.

In summary, we demonstrate herein the effects of miR-219 on OPC proliferation and differentiation, remyelination, and forelimb function deficit in contusion SCI rats. Our work revealed that MCT-1 may be involved in the proliferation and differentiation of OPCs induced by miR-219. This study provides a potential novel therapeutic target for promoting functional recovery after contusion SCI.

## Figures and Tables

**Table 1:** BrdU regimen.

Time points	Sacrifice (dpi)	BrdU regimen
Sham	7	4 h, 1-3 dpi
1 d	1	4 h, 1 dpi
3 d	3	4 h, 1-3 dpi
7 d	7	4 h, 1-3 dpi
10 d	10	4 h, 1-7 dpi
14 d	14	4 h, 1-7 dpi

dpi: days of postinjury.

**Table 2:** Forward and reverse primer sequences for qRT-PCR.

Rat gene	Forward primer (5′-3′)	Reverse primer (5′-3′)
miR-219	ACACTCCAGCTGGGTGATTGTCCAAACGCA	Kit available
U6	GCTTCGGCAGCACATATACTAA	CGAATTTGCGTGTCATCCTT
MCT-1	GCTGCTTCTGTTGTTGCGAATGGA	AAAGGCAAATCCAAAGACTCCCGC
GAPDH	GACATGCCGCCTGGAGAAAC	AGCCCAGGATGCCCTTTAGT

MCT-1: monocarboxylate transporter 1; GAPDH: glyceraldehyde 3-phosphate dehydrogenase.

## Data Availability

The data used to support the findings of this study are available from the corresponding author upon request.

## Abbreviations

miR-219: MicroRNA-219
OPCs: Oligodendrocyte precursor cells
OLs: Oligodendrocytes
As: Astrocytes
LFB: Luxol fast blue
MCT-1: Monocarboxylate transporter 1
GAPDH: Glyceraldehyde 3-phosphate dehydrogenase
SCI: Spinal cord injury
CNS: Central nervous system
RT: Room temperature
MBP: Myelin basic protein
GFAP: Glial fibrillary acidic protein
BrdU: Bromodeoxyuridine
qRT-PCR: Quantitative real-time PCR
PBS: Phosphate-buffered saline.
